# Supplementary material for: Differences in Sources of Information, Risk Perception, and Cognitive Appraisals between People with Various Latent Classes of Motivation to Get Vaccinated against COVID-19 and Previous Seasonal Influenza Vaccination: Facebook Survey Study with Latent Profile Analysis in Taiwan
Source: Vaccines (Basel). 2021 Oct 19;9(10):1203. doi: 10.3390/vaccines9101203 (PMC8538554; doi:10.3390/vaccines9101203)
Supplement: Supplementary file 1 [file vaccines-09-01203-s001.zip › vaccines-1416156-supplementary.pdf]

Advertisement for Recruiting the Participants

大家好:

我們是高雄醫學大學附設醫院的研究人員，誠摯邀請您填寫一份網路調查問卷。由於台灣的衛生福利部已經和國外簽約，將購買研發中用於預防新型冠狀病毒肺炎的疫苗，國內研發單位也積極進行試驗中，所以以下將邀請您利用10分鐘時間填答一份網路問卷，來了解您對於未來可能會供世人施打的新型冠狀病毒肺炎疫苗的看法。

這份調查不必填寫姓名或任何能辨識個人身分的資料，您無須擔心個人資料外流問題。填答結果僅供學術研究分析，可供擬定衛生政策之參考。

如果您願意參加研究，請按下面「願意參加」，接著開始填答問卷；如果不想參加研究，請按「不願意參加」，即可結束此訊息。請仔細閱讀每一題，然後點選最符合您想法和經驗的答案。問卷最後會提供衛生福利部的防疫網站連結，您可從此網站獲得防疫建議，請填答到最後。感謝您！

高雄醫學大學附設中和紀念醫院　顏正芳教授　敬上

Dear everybody

This is a message from the researchers of Kaohsiung Medical University Hospital to invite you to participate into an online survey. Currently, Taiwan' Ministry of Health and Welfare has signed the contracts with foreign firms to import the vaccines against COVID-19. Research and development units in Taiwan are also actively testing new vaccines against COVID-19. We would like to invite you to complete an online questionnaire. This online survey may take you 10 minutes. The purpose of survey is to understand your attitudes toward vaccines against COVID-19.

This is anonymous questionnaire, and your privacy is protected. Your participation will help to make the policies for responding COVID-19 vaccination.

If you agree to participate into this study, please press the button "Agree to participate" and go to the research questionnaire website. If you do not want to participate into this study, please press the button "Disagree to participate" and leave the advertisement. Please answer each item and select the appropriate response. At the end of the online questionnaire, we provide the link to the page established by the Ministry of Health and Welfare. We appreciate your participation.

Professor and Dr. Cheng-Fang Yen, Kaohsiung Medical University Hospital
